# Combined effects of predator cues and competition define habitat choice and food consumption of amphipod mesograzers

**DOI:** 10.1007/s00442-017-4056-4

**Published:** 2018-01-15

**Authors:** Jan Beermann, Karin Boos, Lars Gutow, Maarten Boersma, Ana Carolina Peralta

**Affiliations:** 1 Department of Functional Ecology, Alfred Wegener Institute, Helmholtz Centre for Polar and Marine Research, PO Box 120161, 27515 Bremerhaven, Germany; 2 Alfred Wegener Institute, Helmholtz Centre for Polar and Marine Research, Biologische Anstalt Helgoland, Postbox 180, 27483 Helgoland, Germany; 3 Helmholtz Institute for Functional Marine Biodiversity, Oldenburg, Germany; 4 MARUM-Center for Marine Environmental Sciences, University of Bremen, Leobener Straße 8, 28359 Bremen, Germany; 5 FB2, University of Bremen, Bremen, Germany; 6 Marine Biology Laboratory, Simon Bolivar University, Caracas, Venezuela

**Keywords:** Apparent competition, Trait-mediated interactions, Predator avoidance, Habitat segregation, *Gammarus*

## Abstract

Predation has direct impact on prey populations by reducing prey abundance. In addition, predator presence alone can also have non-consumptive effects on prey species, potentially influencing their interspecific interactions and thus the structure of entire assemblages. The performance of potential prey species may, therefore, depend on both the presence of predators and competitors. We studied habitat use and food consumption of a marine mesograzer, the amphipod *Echinogammarus marinus*, in the presence/absence of a fish mesopredator and/or an amphipod competitor. The presence of the predator affected both habitat choice and food consumption of the grazer, indicating a trade-off between the use of predator-free space and food acquisition. Without the predator, *E. marinus* were distributed equally over different microhabitats, whereas in the presence of the predator, most individuals chose a sheltered microhabitat and reduced their food consumption. Furthermore, habitat choice of the amphipods changed in the presence of interspecific competitors, also resulting in reduced feeding rates. The performance of *E. marinus* is apparently driven by trait-mediated direct and indirect effects caused by the interplay of predator avoidance and competition. This highlights the importance of potential non-consumptive impacts of predators on their prey organisms. The flexible responses of small invertebrate consumers to the combined effects of predation and competition potentially lead to changes in the structure of coastal ecosystems and the multiple species interactions therein.

**Electronic supplementary material:**

The online version of this article (10.1007/s00442-017-4056-4) contains supplementary material, which is available to authorized users.

## Introduction

The global decline in populations of large predatory fish caused by overfishing and over-exploitation of local resources has contributed to an increase in populations of medium-sized predatory fish (10–30 cm) and crustaceans (‘mesopredators’) in marine systems (Myers and Worm 2003; Baum and Worm 2009; Eriksson et al. 2009). This process, referred to as ‘mesopredator release’ (cf. Eriksson et al. 2011), has resulted in a transition from large predator- to mesopredator-dominated systems in many marine areas (Baum and Worm 2009; Eriksson et al. 2011; Ory et al. 2012).

Marine mesograzers (i.e., small herbivorous invertebrates ≤ 2.5 cm) are common prey organisms of mesoedators. Accordingly, many marine mesograzer communities are controlled by top–down processes, depending on the presence and diversity of predators (Douglass et al. 2008; Amundrud et al. 2015). Mesograzers affect macroalgal performance and productivity by grazing on algal thalli (negative effect) or by removing epiphytes (positive effect) (Andersson et al. 2009). Therefore, mesoredators can influence macroalgal performance by controlling mesograzer abundance and thus grazing intensity (Davenport and Anderson 2007; Moksnes et al. 2008; Eriksson et al. 2009; Poore et al. 2014). Apart from the direct effect of predation (i.e., the decrease of prey abundance), indirect effects of the predators on mesograzer species might be equally or even more important. These non-consumptive effects of predator presence can alter interspecific interactions among prey species, thus driving the composition, abundance, and functioning of mesograzer communities (i.e., trait-mediated indirect effects; Paterson et al. 2013; Amundrud et al. 2015).

Shelter from predation could be more pivotal for a species than access to potential food sources and should concentrate animals in protected (micro) habitats (Best and Stachowicz 2012; Gutow et al. 2012; Whalen et al. 2013; Beermann and Boos 2015). Mesograzers often live in close association with seaweeds, and indeed, many species select their algal host for the protection it provides from predators (Buschmann 1990; Duffy and Hay 1991; Lancellotti and Trucco 1993; Sotka 2007; Lasley-Rasher et al. 2011). The aggregation of species in protective habitats may increase competition between mesograzer species and affect habitat use of co-occurring species. Therefore, habitat choice and feeding behavior of mesograzers within their algal refuges (and/or the degree of habitat partitioning among species) might depend on both the presence of predators and competitors.

Amphipod crustaceans are common in algal assemblages and many species are effective mesograzers, exerting strong effects on algae (e.g., Duffy and Hay 2000; Andersson et al. 2009; Poore et al. 2012). Simultaneously, amphipods represent an important food source for predatory fish and larger invertebrates (Edgar and Shaw 1995; Moksnes et al. 2008; Pérez-Matus et al. 2012). For example, the omnivorous amphipod *Echinogammarus marinus* (Leach 1815) is frequently found on north-eastern Atlantic shores. The species inhabits dense algal assemblages in the upper intertidal zone, where it is subject to harsh environmental conditions (i.e., strong variation in salinity and temperature), but where fish predators are naturally less abundant (Vlasblom 1969; Van Maren 1975; Pinkster and Broodbakker 1980; Martins et al. 2014). The sympatric *Gammarus locusta* (Linnaeus 1758) is mostly associated with abundant algae in habitats from the lower intertidal and shallow subtidal zones to floating algal assemblages (Van Maren 1975; Andersson et al. 2009; Gutow et al. 2015). Hence, these species are spatially segregated in the field, but both amphipod species are effective mesograzers with similar preferences for food and habitat under predator- and competitor-free conditions (pers. obs.). The spatial segregation in the field may be a result of competitive exclusion, with *E. marinus* evading interspecific competition with *G. locusta* for predator-free space in favourable habitats. We thus hypothesize that the performance of *E. marinus* and its distribution in the field is driven by non-consumptive effects of predators and interspecific competition with sympatric gammarids. To test this hypothesis, we studied habitat use and food consumption of *E. marinus* in the presence/absence of predators and/or competitors. We first investigated single and combined effects of predator presence and interspecific competitor cues on *E. marinus*. In a second experimental setup, we tested for the effects of direct interference with a competitor and competitor density in the presence/absence of predator cues. The possible habituation of *E. marinus* to constant predator presence was investigated in a third experiment.

## Materials and methods

### Animal collection and maintenance

Approximately 1000 individuals of *Echinogammarus marinus* were collected in the rocky intertidal zone of the island of Helgoland (German Bight, North Sea; 54º11′21′′N; 7º52′60′′E) in June and July 2015. Amphipods were collected from under flat rocks covered with canopies of the brown seaweed *Fucus vesiculosus* Linnaeus 1753. The specimens were maintained for 1 month under stable environmental conditions (flow-through of filtered seawater, average temperature of 16.5 ± 0.5 °C, natural ambient light conditions) in a large aquarium ensuring a stock of animals large enough for experiments. The amphipods were fed thalli of *F. vesiculosus*. Individuals of *Gammarus locusta* were taken from a laboratory culture in a large aquarium under the exact same conditions as described above for *E. marinus.*

Approximately 50 individuals of the sea scorpion *Taurulus bubalis* (Euphrasen 1786) were collected with a bow net in Helgoland Harbor. The fish were kept in three separate aquaria (50 × 100 × 45 cm) for 2 weeks under stable environmental conditions (15.0 ± 0.5 °C; 12 h light: 12 h darkness) in a flow-through system with filtered seawater. Until being used in the experiments, the fish were fed a diet of live *E. marinus*, which were actively chased and eagerly eaten by the fish.

### General experimental setup

**Fig. 1:**
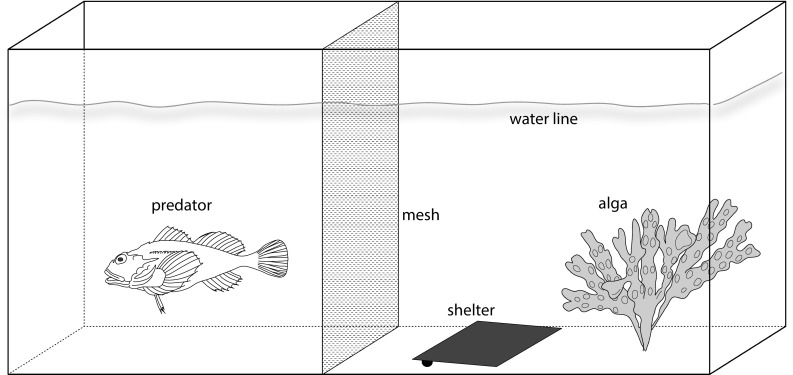
General experimental setup for the investigation of habitat choice and food consumption in the amphipod *Echinogammarus marinus*

The following setup was employed for all experimental treatments: small transparent plastic aquaria (8 l; 32.5 × 17.5 × 18.5 cm) were each supplied with a uni-directional flow-through system of filtered seawater of approx. 800 mL/min. All experiments were conducted under constant laboratory conditions (average temperature of 16.5 ± 0.5 °C; 12 h light: 12 h darkness). Each aquarium was divided in half by a plastic mesh (mesh size 1 mm). The posterior half of the aquarium, opposite to the entry of the water flow, was supplied with a thallus of the macroalga *F*. *vesiculosus* (biomass: 700 mg) and a slightly sloping ceramic tile (10 × 10 cm), providing a hiding place similar to flat stones in the field (Fig. 1). To test the influence of the presence of a potential predator, a single specimen (ca. 10 cm body length) of *T. bubalis* was placed in the anterior half of the aquarium. The seawater flow was directed from the fish towards the amphipods. During the experiment, the fish were not fed, preventing that cues of wounded conspecifics may have affected the behavior of the amphipods.

In all experiments, groups of 20 individuals of *E. marinus* of similar size (18 ± 2 mm body length) were placed in the aquaria and starved for 24 h to standardize hunger levels among individuals. The experiments were then started with the addition of the algae and, where applicable, the individual predators to the setups. After 24 h, the position of each individual amphipod was assessed at daytime without manipulating the container. Five different habitats were distinguished: (1) on the algal thallus; (2) under the tile; (3) clinging to the mesh that separated the aquarium; (4) attached to a wall of the aquarium, touching the water surface, and/or leaving the water partly or completely; and (5) any remaining part of the aquarium. Dead individuals were recorded.

After the non-manipulative assessment, the algae were removed from the setups and their wet weights were measured. The resulting feeding rates in the different experimental setups were calculated by the following equation (see Cronin and Hay 1996):

$${\text{feeding rate}} = \left[ {\left( {{\text{Wi}} \times \frac{\text{Cf}}{\text{Ci}}} \right) - {\text{Wf}}} \right] \times {\text{day}}^{ - 1} .$$

Initial (Wi) and final weight (Wf) of the thallus were measured before and after each experiment. For each experiment, controls without any animals were run in five separate aquaria (cf/ci) to control for autogenic weight change of the algae. All weights were measured by blotting the thalli on laboratory paper to remove any surface water.

Five replicates (aquaria) were conducted per treatment for each experimental setup. Each amphipod and fish predator was used only once.

### Presence of a predator and interspecific competitors

In a first experiment, food consumption and habitat utilization of *E. marinus* were tested in the presence and absence of the fish predator *T. bubalis* and the competitor *G. locusta* in a full-factorial design. Groups of five individuals of *G. locusta* (13 ± 1 mm body length) were placed in small cages without food (7 × 5 × 5 cm). One cage was deployed in each aquarium in the same halves as *E. marinus*. The cages clearly separated *G. locusta* from the experimental animals, thus preventing direct interference between hetero-specific individuals. After 24 h, habitat utilization and feeding rates were evaluated as described above.

### Direct interference effect

A second experiment tested for the effects of direct interference on habitat choice and food consumption of *E. marinus* in the presence of the predator *T. bubalis*. Three different treatments, with groups of 5, 10, and no individuals of *G. locusta* (same size class: 13 ± 1 mm), were placed without cages, thus allowing for direct encounter between individuals of the two species. After 24 h, the position of each *E. marinus* was registered. As both species were feeding on the same algal thalli and the group sizes were unequal, per capita feeding rates were calculated for both single-species and mixed-species setups by dividing the overall food consumption by the total number of amphipods (*E. marinus* and *G. locusta*) employed in the respective setups.

### Habituation to predator presence

A third experiment tested for possible habituation of *E. marinus* to the fish cue stimulus. Habitat utilization and food consumption of *E. marinus* were measured as described above every 24 h over a total period of 96 h. After each measurement, both the algal thalli and the *T. bubalis* individuals were replaced by new specimens. This minimized possible aging effects of the algae, ensured a steady food supply for the amphipods, and prevented any negative effects on the fish that could have also affected the amphipods’ behavior. Dead individuals of *E. marinus* were not replaced.

### Statistical analyses

Two response variables (‘habitat choice’ and ‘food consumption’) were measured in each of the three experiments. For the first experiment (‘predator and competitor cues’), a multivariate approach was used to analyze the response variable ‘habitat choice’. We modelled the individual habitats as counts (i.e., shares) of individuals in multiple categories (i.e., ‘shelter’, ‘algae’, ‘mesh’, ‘waterline’, and ‘remaining areas’) with multinomial regression analyses. This type of analyses was most appropriate, because it accounts for the dependent nature of the data in multiple choice designs. Furthermore, the respective sizes of the habitats each provided sufficient space to harbor much more than all employed animals at once, thus ensuring that the individuals could perform choice behaviors independent of animal density. In addition, we found no indication for gregarious behavior in preliminary experiments. Occasionally, individuals were found dead after an experimental trial. In those cases, the number of perished animals was modelled for technical reasons as an additional choice category. The multivariate model included the fixed factor ‘predator’ with the two levels absent and present. In addition, the model for the first experiment included the fixed factor ‘competitor’ with two levels (absent and present). The model included the interaction between the two main terms.

The response variable ‘food consumption’ was analyzed using a general linear model (LM) with a fully crossed two-way design. The predictor variables in this univariate model were the same as for the habitat choice analyses, including the interaction.

To confirm the assumptions of normally distributed and homogenous residuals, qq plots and the residuals plotted against the fitted values were visually inspected (Quinn and Keough 2002). In cases where the assumptions were not met, the data were log transformed to fulfill the criteria.

To establish the significances of individual terms (interaction terms and main factors), likelihood ratio tests (LRT; using F tests in the fixed models and Chi^2^ tests in the mixed model) were used to compare the residual sum of squares of the respective full models with those of the corresponding reduced models not comprising the respective factor and/or term of interest. For intuitive interpretations of the response variable ‘habitat choice’, we calculated the predicted probabilities (fitted values in %) for each category outcome (i.e., habitat type) across predictor levels (i.e., absence or presence of the predator and/or competitor, respectively). This was done using the ‘predict’ function (in the software R; see below) on the individual model coefficients for all combinations.

For the second experiment (‘direct interference’), the response variable ‘habitat choice’ was analyzed using the same multivariate approach as for the first experiment including the fixed factor ‘competitor’ with three abundance levels (absent, 5 individuals, and 10 individuals). The response variable ‘food consumption’ was analyzed using the same general linear model (LM) with a fully crossed two-way design as in the first experiment.

For the third experiment (‘habituation’), the response variable ‘habitat choice’ was analyzed using the same multivariate model as for the first and second experiments. ‘Time’ was included as continuous fixed effects factor into the model and a random within-subjects factor was incorporated as a random term, where replicate was nested in time. For the interpretations, we averaged the predictions for all replicates. The response variable ‘food consumption’ was analyzed using a general linear mixed model (LMM) with a repeated measures design (within-subjects factor). The predictor variables in the univariate model were the same as described before, including the interactions and with the additional random within-subjects factor. To establish the significances of individual terms (interaction terms and main factors), LRTs using Chi^2^ tests for both the multivariate model and the mixed model were applied.

All models were fitted in R, version 3.2.2 (R Development Core Team 2015), using the generic function ‘lm’, the ‘lmer’ function from the ‘lme4’ package (Bates et al. 2015) for the food consumption analyses and the ‘multinom’ function from the ‘nnet’ package (Venables and Ripley 2002) for the habitat choices.

## Results

### Presence of a predator and interspecific competitors

**Fig. 2:**
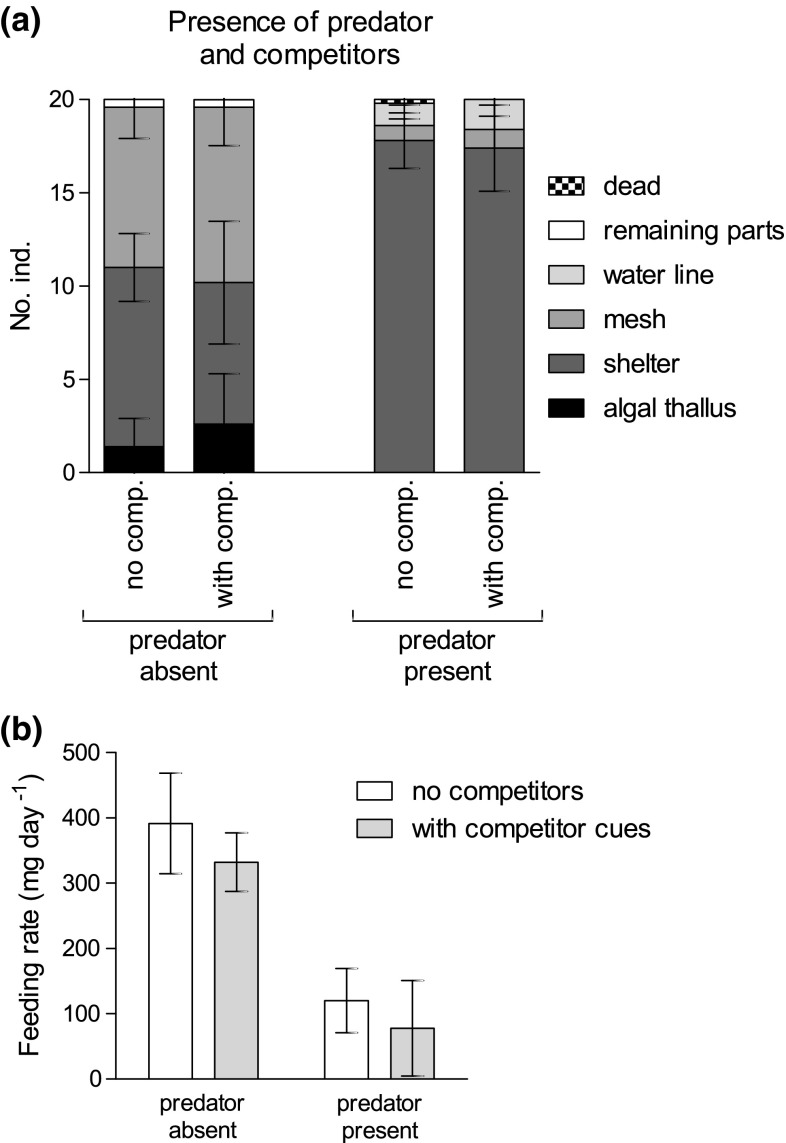
Spatial distribution (**a**) and food consumption (**b**) of groups of 20 individuals each of *Echinogammarus marinus* in the presence of the predator *Taurulus bubalis* and with cues of the congeneric competitor *Gammarus locusta*. Note that only a single dead individual was found; mean ± SD (each *N* = 5)

The comparisons of the animals’ habitat utilizations revealed no interaction of the factor ‘predator’ and the presence of a competitor (LRT_competitor:predator_: *χ*^2^ (5) = 0.0108; *p* = 0.9999). Similarly, the presence of competitors alone did not affect animals’ habitat choice (LRT_competitor_: *χ*^2^ (5) = 4.9724; *p* = 0.4192). However, the presence of the fish predator had a clear effect on habitat utilization of *Echinogammarus marinus* (LRT_predator_: *χ*^2^ (5) = 163.2095; *p* < 0.0001), independent of the presence of the competitor. In the absence of predators, the combined predicted probability of finding individuals on the mesh or on the algal thallus was 43%, whereas the probability to find animals in the shelter was 47%. In the presence of a predator, however, the amphipods were largely confined to the shelter below the tile (probability: 90%) with a low but increased probability of 6% to be found near the water line (Fig. 2a).

The two factors also showed no significant interactive effect on the consumption rate (LRT_competitor:predator_: *F*_1,16_ = 0.0854; *p* = 0.7738). The presence of the predator alone, however, had a strong effect on food consumption of the amphipods (LRT_predator_: *F*_1,18_ = 92.9360; *p* < 0.0001), which clearly decreased when the predator was present in both trials (Fig. 2b). The average feeding rates were slightly lower in the presence of the competitor, although this was not statistically significant (LRT_competitor_: *F*_1,18_ = 3.4675; *p* = 0.0799). Thus, the presence of predators affected behavior and grazing, whereas the presence of competitors did not.

### Direct interference effect

**Fig. 3:**
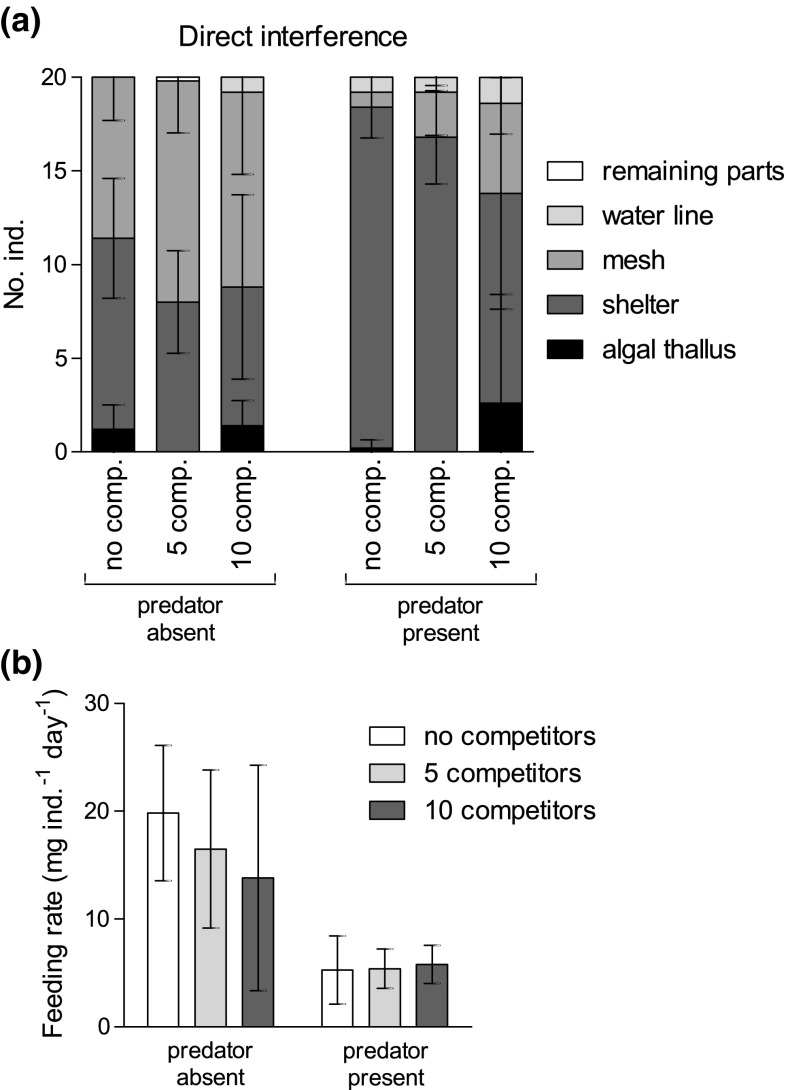
Spatial distribution (**a**) and *per capita* food consumption (**b**) of groups of 20 individuals each of *Echinogammarus marinus* in direct interaction with groups (5 or 10 individuals each) of *Gammarus locusta* and in the presence of the predator *Taurulus bubalis*; mean ± SD (each *N* = 5; no mortality occurred). Note that for the single-species trials (without competitor), the consumption rates were calculated for *E. marinus* only, whereas for the mixed-species trials (with the competitor *G. locusta*), the combined consumption rates were calculated considering the individuals of both species

In the presence of a predator and in direct interference with the potential competitor, there was a significant interaction in the habitat use of *E. marinus* [LRT_competitor:predator_: *χ*^2^ (8) = 20.5699; *p* = 0.0083]. Moreover, the animals’ habitat choice, again, strongly depended on the presence of the fish predator [LRT_predator_: *χ*^2^ (4) = 112.0918; *p* < 0.0001] and on the direct contact with the competitor [LRT_competitor_: *χ*^2^ (8) = 56.1650; *p* < 0.0001]. As before, in the presence of the predator, the amphipods had a stronger preference for the shelter in all trials. However, the predicted probabilities for finding animals in the shelter decreased with increasing competitor density from 91% (competitor absent) to 56% (10 competitors) (Fig. 3a). At the same time (i.e., in the presence of the predator), the combined probabilities to find *E. marinus* on the mesh or on the algal thallus increased from 5% (competitor absent) to 12% (5 competitors) and even 37% (10 competitors). In the trials, *G. locusta* was mainly found in the shelter, but never on the algal thallus (see Fig. S1 in the Electronic Supplement).

In the absence of a predator, the amount of algal tissue that was consumed by *E. marinus* (single-species trial) or both species (in the two mixed-species trials), respectively, slightly decreased on average with increasing density of the competitor *G. locusta*, whereas feeding rates remained similarly low for all competitor densities when a predator was present (Fig. 3b). However, due to high variability in the data, the differences in feeding rates were statistically not significant (LRT_competitor:predator_: *F*_2,24_ = 1.3127; *p* = 0.2877), also leading to no effect of competition (LRT_competitor_: *F*_2,27_ = 0.5366; *p* = 0.5911). The presence of a predator clearly reduced the food consumption in all treatments (LRT_predator_: *F*_1,28_ = 26.4800; *p* < 0.0001). Thus, habitat choice was affected by the actual presence of competitors, but this was not reflected in the combined consumption rates of the two species.

### Habituation to predator presence

**Fig. 4:**
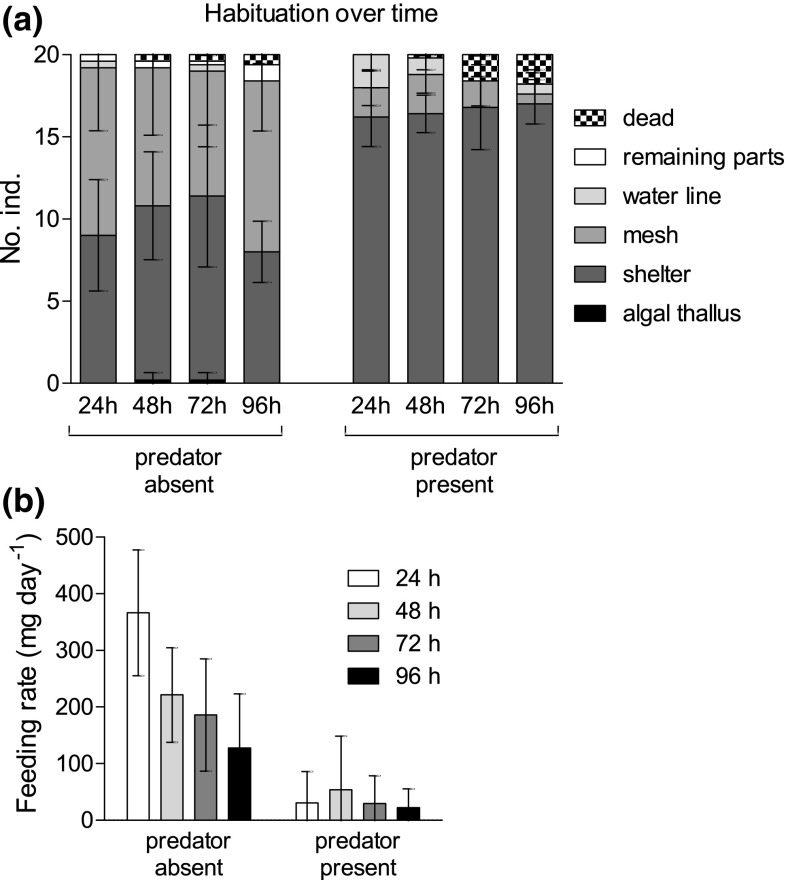
Spatial distribution (**a**) and food consumption (**b**) of groups of 20 individuals each of *Echinogammarus marinus* over the consecutive duration of 24, 48, 72, and 96 h in the presence of the predator *Taurulus bubalis*; mean ± SD (each *N* = 5)

The reaction in habitat choice to predator cues was independent of time over the entire experimental period of 96 h [LRT_time:predator_: *χ*^2^ (5) = 4.0488; *p* = 0.5424]. The amphipods responded to the predator cue treatment [LRT_predator_: *χ*^2^ (5) = 185.7960; *p* < 0.0001] and the general patterns remained the same over time (Fig. 4a): in the absence of the predator, the predicted probabilities to find individuals in the shelter or on the mesh were similar (between 47–49 and 42–48%, respectively), whereas probabilities to find individuals in the shelter remained high (81–84%) when the predator was present. However, the increase in dead individuals (Fig. 4a) over the duration of the experiment (1.8 ± 1.09 individuals after 96 h) resulted in a significant effect of the main factor ‘time’ [LRT_time_: *χ*^2^ (5) = 24.8737; *p* < 0.0001].

In the absence of a predator, feeding rates decreased over time, whereas the consumed amounts in the presence of a predator were consistently low [LRT_time:predator_: *χ*^2^ (1) = 7.2290; *p* = 0.0071; Fig. 4b]. Overall, the feeding rate was consistently lower in the presence of the fish predator [LRT_predator_: *χ*^2^ (1) = 13.9900; *p* = 0.0002].

See the Electronic Supplementary Data (Tables S1, S2) for the full results of the statistical analyses.

## Discussion

### Predator presence

The presence of a fish mesopredator clearly affected both habitat choice and feeding activity of *Echinogammarus marinus*, indicating a trade-off between shelter and food acquisition. In the absence of the predator, some amphipods were found associated with the algae which served as food for the animals. In contrast, in the presence of a predator and in the absence of direct interference with competitors, the individuals completely avoided the algal thalli and hid below the ceramic tile, where, under field conditions, they would probably be less easily accessible for larger fish predators. Apparently, the amphipods preferred shelter over food under the risk of predation. In the current setups, however, the habitat choice of the animals was evaluated at daytime only. Feeding and possible behavioral responses to predator cues probably differ in their level of intensity and manifestation during night time (Szokoli et al. 2015). Amphipods probably leave protective habitats preferably at night to forage on food algae (Buschmann 1990). Accordingly, the observed patterns of habitat use may be an underestimation of the actual use of the algal habitat by *E. marinus*. The obtained consumption of algal biomass indicates a frequent and extensive association with the algal habitat at night when the amphipods are protected from visually hunting predators. However, food consumption of the amphipods decreased in the presence of the predator. This response has also been observed in other marine amphipods (Reynolds and Sotka 2011; Reynolds and Bruno 2013). The reduced feeding activity may be the consequence of spatial segregation from the food source, because the animals spent more time under the protective tile (Lima and Dill 1990), or a direct behavioral response to the predator presence (Alexander et al. 2013).

Common reactions to predator cues by aquatic crustaceans involve the search for refuge (i.e., complex structures, shelter), reduction of activity, association with chemically defended algae, withdrawal from the predator, and/or emigration to predator-free patches (e.g., Thiel and Reise 1993; Abjörnsson et al. 2000; Lindén et al. 2003; van Son and Thiel 2006; Zamzow et al. 2010; Beermann and Boos 2015). In contrast to our current findings, *E. marinus* did not change its habitat use, but reduced its movement activity in experiments with a different fish mesopredator, the shanny *Lipophrys pholis* (Alexander et al. 2013). This indicates predator-specific avoidance behaviors (e.g., McIntosh and Peckarsky 1999; Abjörnsson et al. 2000; van Son and Thiel 2006). Similarly, the avoidance behavior of the amphipods in our experiments could be a specific reaction to the particular cues released by *Taurulus bubalis*. Some amphipods respond to the presence of benthic predators with emigration (e.g., Thiel and Reise 1993; van Son and Thiel 2006). The role of emigration in the predator avoidance of *E. marinus* cannot be evaluated from our laboratory setups, where the amphipods had no opportunity to emigrate. The presence of fish, however, mostly induces use of sheltered (micro)habitats that are less accessible to these predators (Thiel and Reise 1993; Abjörnsson et al. 2000; van Son and Thiel 2006; Beermann and Boos 2015), indicating that hiding in a sheltered habitat may likely represent the primary response of *E. marinus* to the presence of *T. bubalis*. Alternatively, an enhanced presence of the amphipods near the waterline or in the remaining parts of the container probably could be an indication for an attempted emigration. In natural habitats, further parameters, such as habitat quality and competition, may also influence prey reactions along with other cues such as signals of actual predation (i.e., cues of wounded conspecifics; Baumgärtner et al. 2003; Pennuto and Keppler 2008; Beermann and Boos 2015).

Some peracarid crustaceans have been shown to habituate to a constant stimulus of fish cues (e.g., Holomuzki and Hatchett 1994). However, the reaction of *E. marinus* to the predator did not change over the entire duration of 4 days. This supports the conclusion of Patterson et al. (2013) from a meta-analysis that both the intensity and the exposure time to fish cues have only little to no effect on the overall prey response. The duration of the current experiment may have been too short to observe long-term habituation to the stimulus as suggested by experiments of longer duration on freshwater amphipods (see Abjörnsson et al. 2000). A flexible response to predator presence may be an important adaptation to changing environmental conditions and/or constant threats of predation (Beermann and Boos 2015). The spatial distribution of *E. marinus* in the field (i.e., under stones and rocks in the intertidal) could thus be a response to the constant stimulus of predator cues, whereas in the laboratory, the animals did not seem to seek for such habitats in the absence of predators.

Interestingly, the feeding rates of *E. marinus* decreased over the 4 days in the predator-free setups of the third experiment, whereas in the presence of the predator, the feeding rates were consistently low. This decrease may reflect a shift from excessive feeding after the initial starvation period at the beginning of the experiment towards a more normal feeding behavior when the animals had unlimited access to food.

### Competitor presence

The direct presence of a competitor affected the habitat choice of the amphipods. In mixed-species trials together with *Gammarus locusta*, *E. marinus* was less likely to be found in the shelter as compared to the single-species trials. This was particularly pronounced in the presence of predator cues. In addition, food consumption was slightly lower in the presence of the competitor or even when *E. marinus* was just exposed to competitor cues. The mixed-species setups did not allow discriminating between the specific consumption rates of *E. marinus* and *G. locusta*. The pooled consumption rates of the two species must, therefore, be regarded with caution as one of the two species may have consumed on average much more algal material than the other. The slightly reduced combined food consumption of the amphipods in the presence of interspecific competitors, however, was not the result of competition for food between the two amphipod species, because algal food was not limited in the experiments. Similarly, natural mesoherbivore communities inhabit extensive macroalgal beds which provide plenty and diverse food for numerous small consumers (Taylor 1998). Alternatively, the reduced consumption may be a direct effect of interspecific interference competition (e.g., Persson 1986). Furthermore, predator presence may induce ‘apparent competition’ among species (also called ‘competition for enemy-free space’; Holt 1977) which has similar implications as competition for food resources. Interference between the animals could, therefore, have hindered the amphipods from feeding, even displacing *E. marinus* to less protected habitats under the threat of predation. This is corroborated by the observed microhabitat preferences of *G. locusta* in the experiments that clearly preferred the shelter over the other habitats in the container (Fig. S1 in the Electronic Supplement).

Persistently strong competition for shelter due to an elevated predation risk may result in competitive exclusion, unless mechanisms such as habitat partitioning reduce the intensity of competition, thereby allowing for a coexistence of species (Schoener 1974, 1986). This type of habitat partitioning is most likely to evolve among frequently co-occurring species. Accordingly, animal species respond flexibly to the presence of competitors (e.g., Ebersole 1985; Beermann and Boos 2015) or exhibit predetermined preferences, leading to a partitioning of resources (e.g., Ebersole 1985; Lürig et al. 2016). Apparently, competition for shelter increased among the competitors (i.e., increased ‘apparent competition’). The observed distributional patterns thus indicate that (1) *G. locusta* is competitively superior, as it displaced individuals of *E. marinus* from the shelter that was preferred by both species (c.f. Jermacz et al. 2015) and/or (2) *E. marinus* is able to respond flexibly to reduce competition with other gammarids. Consequently, under natural conditions, an apparent competition probably results in a higher predation risk for *E. marinus*. This is corroborated by the distribution pattern of the two species in the field: *G. locusta* occupies a wider range of habitats, whereas *E. marinus* is mainly restricted to intertidal and euryhaline habitats, where the species may have a competitive advantage due to its higher tolerances to harsh environmental conditions (Van Maren 1975; Pinkster and Broodbakker 1980).

## Conclusions

Trait-mediated indirect interactions with mesopredators may either be positive (mesograzer activity reduction) or negative for certain algae species (habitat shift of mesograzers to particular algal species) (Schmitz et al. 2004). Simultaneously, intensity and nature of intraguild interactions may largely depend on the degree of predation pressure, because an increased aggregation of prey species at ‘safe’ sites likely modifies competitive relationships (i.e., trait-mediated indirect interactions; Holt 1984; Pallini et al. 1998; Werner and Peacor 2003). In our model system, the performance of *E. marinus* is determined by trait-mediated direct and indirect effects caused by the presence of predators and competitors. As the fish presence affected the food consumption of the amphipods, the presence of mesoredators should have a positive trait-mediated indirect effect on algae in addition to the direct effects of predation on the mesograzers. Consequently, a mesopredator release can have strong non-consumptive impacts on mesograzer performance. Substantial changes in the structure and composition of marine species assemblages in coastal ecosystems could be a consequence.

## Electronic supplementary material

Below is the link to the electronic supplementary material.

Supplementary material 1 (PDF 140 kb)
