# Supplementary material for: Combined effects of predator cues and competition define habitat choice and food consumption of amphipod mesograzers
Source: Oecologia. 2018 Jan 15;186(3):645–54. doi: 10.1007/s00442-017-4056-4 (PMC5829112; doi:10.1007/s00442-017-4056-4)
Supplement: Supplementary file 1 — Supplementary material 1 (PDF 140 kb) [file 442_2017_4056_MOESM1_ESM.pdf]

Combined effects of predator cues and competition define habitat choice and food consumption of amphipod mesograzers

- Electronic Supplementary Material -

Jan Beermann[1,2,3]*, Karin Boos[4], Lars Gutow[1], Maarten Boersma[2,5], Ana Carolina Peralta[2,6]

[1]Alfred Wegener Institute, Helmholtz Centre for Polar and Marine Research; Functional Ecology; PO Box 120161, 27515 Bremerhaven, Germany

[2]Alfred Wegener Institute, Helmholtz Centre for Polar and Marine Research; Biologische Anstalt Helgoland; Postbox 180, 27483 Helgoland, Germany

[3]Helmholtz Institute for Functional Marine Biodiversity, Oldenburg, Germany

[4]MARUM - Center for Marine Environmental Sciences, University of Bremen, Leobener Straße 8, 28359 Bremen, Germany

[5]University of Bremen; FB2; Bremen, Germany

[6]Simon Bolivar University, Marine Biology Laboratory, Caracas, Venezuela

*corresponding author, email address: Jan.Beermann@awi.de

Table S1. Summary of results of the multinomial regression analyses for the response variable 'habitat choice' (i.e. counts of individuals in multiple categories).

Habitat choice

				**Factor levels**		**% predicted probability of appearance**					
**Experiment 1**	df	Chi	p	predator	competitor	shelter	algae	mesh	waterline	remaining areas	dead
LM: $LRT_{predator*competitor}$	5	0,01084	0,999999	absent	absent	48	7	43	0	2	0
LM: $LRT_{predator}$	5	163,2095	**< 0.0001**	present	absent	89	0	4	6	0	1
LM: $LRT_{competitor}$	5	4,972489	0,419247	absent	present	38	13	47	0	2	0
				present	present	87	0	5	8	0	0
				**Factor levels**		**% predicted probability of appearance**					
**Experiment 2**	df	Chi	p	predator	competitor	shelter	algae	mesh	waterline	remaining areas	dead
LM: $LRT_{predator*competitor}$	8	20,56998	**0,008382**	absent	absent	51	6	43	0	0	-
LM: $LRT_{predator}$	4	112,0918	**< 0.0001**	absent	5 ind.	40	0	59	0	1	-
LM: $LRT_{competitor}$	8	56,16506	**< 0.0001**	absent	10 ind.	37	7	52	4	0	-
				present	absent	91	1	4	4	0	-
				present	5 ind.	84	0	12	4	0	-
				present	10 ind.	56	13	24	7	0	-
				**Factor levels**		**% predicted probability of appearance**					
**Experiment 3**	df	Chi	p	predator	time	shelter	algae	mesh	waterline	remaining areas	dead
LM: $LRT_{predator*competitor}$	5	4,048881	0,5424	absent	24 h	50,0	0,5	45,7	1,7	1,4	0,7
LM: $LRT_{predator}$	5	185,796	**< 0.0001**	absent	48 h	49,4	0,5	46,0	1,1	2,0	1,1
LM: $LRT_{competitor}$	5	24,87372	**0,000147**	absent	72 h	48,5	0,5	45,9	0,7	2,8	1,7
				absent	96 h	47,2	0,5	45,4	0,5	3,8	2,5
				present	24 h	78,8	0,0	11,4	9,1	0,0	0,6
				present	48 h	84,3	0,0	9,0	5,0	0,0	1,8
				present	72 h	86,1	0,0	6,8	2,6	0,0	4,5
				present	96 h	82,9	0,0	4,8	1,3	0,0	11,1

Table S2. Summary of LM results for the response variable 'food consumption' (i.e. feeding rates).

Food consumption				
**Experiment 1**	df	F	p	
LM: $LRT_{predator*competitor}$	16,1	0,0854	0,7738	
LM: $LRT_{predator}$	18,1	92,936	**< 0.0001**	
LM: $LRT_{competitor}$	17,1	3,4675	0,07997	
**Experiment 2**	df	F	p	
LM: $LRT_{predator*competitor}$	24,2	1,3127	0,2877	
LM: $LRT_{predator}$	26,1	26,48	**< 0.0001**	
LM: $LRT_{competitor}$	26,2	0,5366	0,5911	
**Experiment 3**	df	Chi	p	
LM: $LRT_{predator*time}$	1	7,229	**0,007173**	
LM: $LRT_{predator}$	1	13,399	**0,000252**	
LM: LRT_{time}	1	5,1692	**0,02299**	not mentioned in text

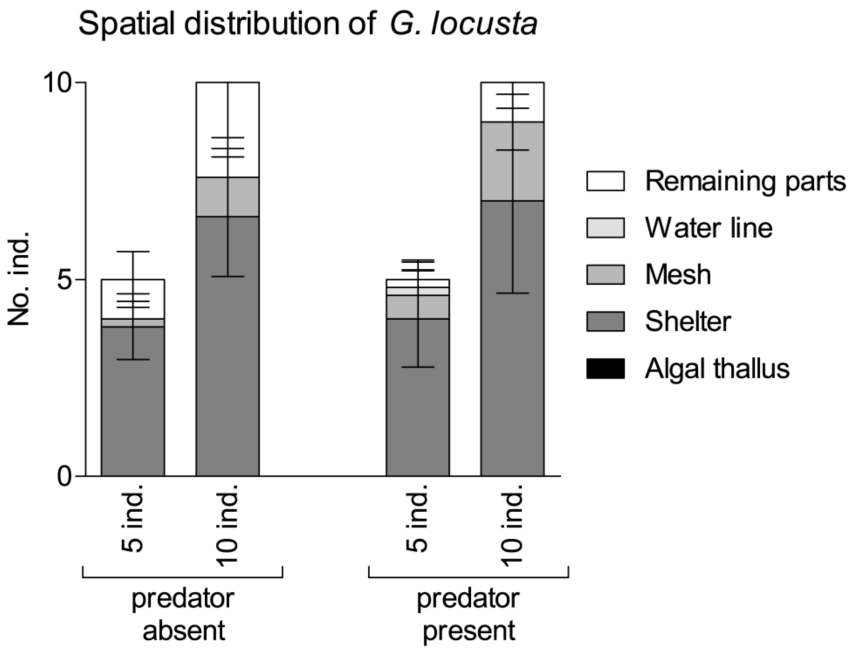

Figure S1. Spatial distribution of the groups (5 or 10) of *Gammarus locusta* in presence/absence of the fish predator *Taurulus bubalis* in the second experiment.
